# Characterization of Italian honeys (Marche Region) on the basis of their mineral content and some typical quality parameters

**DOI:** 10.1186/1752-153X-1-14

**Published:** 2007-06-07

**Authors:** Marcelo Enrique Conti, Jorge Stripeikis, Luigi Campanella, Domenico Cucina, Mabel Beatriz Tudino

**Affiliations:** 1 SPES Development Studies Research Centre, Università di Roma "La Sapienza", Rome, Italy; 2 INQUIMAE, Departamento de Química Inorgánica, Analítica y Química Física, Facultad de Ciencias Exactas y Naturales, Universidad de Buenos Aires, Buenos Aires, Argentina; 3 Dipartimento di Chimica, Università di Roma "La Sapienza", Rome, Italy

## Abstract

**Background:**

The characterization of three types of Marche (Italy) honeys (Acacia, Multifloral, Honeydew) was carried out on the basis of the their quality parameters (pH, sugar content, humidity) and mineral content (Na, K, Ca, Mg, Cu, Fe, and Mn). Pattern recognition methods such as principal components analysis (PCA) and linear discriminant analysis (LDA) were performed in order to classify honey samples whose botanical origins were different, and identify the most discriminant parameters. Lastly, using ANOVA and correlations for all parameters, significant differences between diverse types of honey were examined.

**Results:**

Most of the samples' water content showed good maturity (98%) whilst pH values were in the range 3.50 – 4.21 confirming the good quality of the honeys analysed. Potassium was quantitatively the most relevant mineral (mean = 643 ppm), accounting for 79% of the total mineral content. The Ca, Na and Mg contents account for 14, 3 and 3% of the total mineral content respectively, while other minerals (Cu, Mn, Fe) were present at very low levels. PCA explained 75% or more of the variance with the first two PC variables. The variables with higher discrimination power according to the multivariate statistical procedure were Mg and pH. On the other hand, all samples of acacia and honeydew, and more than 90% of samples of multifloral type have been correctly classified using the LDA. ANOVA shows significant differences between diverse floral origins for all variables except sugar, moisture and Fe.

**Conclusion:**

In general, the analytical results obtained for the Marche honeys indicate the products' high quality. The determination of physicochemical parameters and mineral content in combination with modern statistical techniques can be a useful tool for honey classification.

## Background

The Community Directive [1] establishes the general definition of honeys that can be marketed in the European Union. The Directive also indicates general and specific compositional characteristics of honey such as sugar content, humidity, acidity, electrical conductivity, diastase activity and hydroxymethylfurfural (HMF) content. Furthermore, labels on honey packaging may be supplemented to include information on the product's regional or topographical origin, floral or vegetable origin, or even specific quality criteria.

Honey is defined as "the natural sweet substance produced by *Apis mellifera *bees from the nectar of plants or from secretions of living parts of plants or excretions of plant-sucking insects on the living parts of plants, which the bees collect, transform by combining with specific substances of their own, deposit, dehydrate, store and leave in honeycombs to ripen and mature" [1]. The beneficial characteristics of honey are its high nutritional value (330 kcal/100 g) and the fast absorption of its carbohydrates on consumption. Moreover, honey exhibits anti-bacterial and anti-inflammatory properties in the treatment of skin wounds and many gastrointestinal diseases [2-5]. This is due to honey's high osmotic pressure, acidity and the hydrogen peroxide content [3,4]. Hydrogen peroxide produced enzymatically is responsible for honey's antibacterial activity.

Italy has the highest number of honey varieties in Europe: 32 unifloral and different varieties of multifloral honeys are produced from a total of 1.070.262 hives [6]. In 2004, honey production reached about 10.200 tons/yr. At present, Italian honeys are strongly affected by competition from Argentine and Chinese varieties whose prices are lower by roughly 50%. Very scarce data are available regarding honey production in the Marche region because of the strong amatorial characteristic of such production and there is some uncertainty in the evaluation of production levels. The commonest honeys produced in the Marche region are multifloral (*Millefiori*) and acacia honeys (unifloral, *Robinia pseudoacacia*). The 2004 production levels in the Marche region were roughly 15 and 20 kg/hive for multifloral and acacia honeys respectively, with a total of 38.000 hives and 209.000 tons of total produced honey [6].

Usually, honey is considered unifloral when the pollen frequency of one plant is over 45% [7]. For honey samples with under-represented pollen grains (i.e. Lavender, Citrus and Rosemary), botanical classification may be achieved with a percentage pollen frequency of only 10–20% [8-14].

Melissopalynology, identification and quantification of pollen grains contained in honey, have together been the traditional method used to ascertain the botanical origin of honeys [7,15,16], but this technique has some limitations [17-22]. A particular difficulty is that melissopalynology requires previous knowledge of pollen morphology and specialised professional personnel to achieve reliable results [23]. However, nowadays in spite of these problems melissopalynology remains the reference method.

The composition and properties of a particular honey sample depend highly on the type of flowers visited by the bees, as well as on the climatic conditions in which the plants grow [24-26]. Honeybees and their products can also be employed as potential bioindicators of environmental contamination [27]. These specific chemical and physical properties may be used for the determination of the botanical origin of honey [18,28-30] and to confirm the results of microscopical analysis.

In recent decades several studies have evaluated some chemical and physicochemical components of honeys, in addition to attempting to establish representative ranges of some of these parameters that would unequivocally determine botanical origin. In characterising unifloral honeys, many authors [11,18,20,22,25,31-37] have suggested the use of physicochemical parameters (i.e. pH, sugar content, electrical conductivity, proline, enzymatic activity, water content, ash content, diastase activity, free and lactonic acidities, etc.) and mineral content (K, Ca, Na, Mg, Fe, etc.) complemented by pollen analysis.

The goal of the present work was first, to verify some of the qualitative parameters such as pH, sugar content and humidity, and second, to contribute to the very scarce available data on mineral content of Marche Region honeys. The elements assessed were: Na, K, Ca, Mg, Fe, Cu and Mn. Furthermore, we have evaluated if the physicochemical parameters and mineral content of Marche honeys can determine the botanical origin. The sampling protocol was made up in order to obtain the most representative insight of the sampled regional areas. All samples were collected in the Montefeltro region, in the Pesaro – Urbino province, a relevant production zone for many typical food products of the Marche region.

## Results and discussion

Table 1 reports the mineral content and physicochemical parameters of honey samples taken from the Marche Region. The mean, standard deviation and the variable ranges are reported according to their botanical origin.

**Table 1:** Descriptive statistics for physicochemical parameters and mineral content (μg g^-1 ^dry weight) in Marche honey samples.

Variable		N	Mean	Dev. Stand.	Min	Max
Sugar	Acacia	23	81.51	0.74	80.20	82.90
	Multifloral	44	80.96	1.41	77.60	83.80
	Honeydew	2	81.70	0.71	81.20	82.20
	Total	69	81.16	1.23	77.60	83.80
pH	Acacia	23	3.68	0.09	3.50	3.82
	Multifloral	44	3.68	0.14	3.51	4.09
	Honeydew	2	4.17	0.06	4.13	4.21
	Total	69	3.70	0.15	3.50	4.21
Moisture	Acacia	23	17.09	0.74	15.70	18.40
	Multifloral	44	17.65	1.41	15.00	21.00
	Honeydew	2	17.10	0.42	16.80	17.40
	Total	69	17.45	1.23	15.00	21.00
Na	Acacia	23	12.86	5.05	6.10	26.40
	Multifloral	44	28.83	8.85	14.10	57.20
	Honeydew	2	62.45	0.07	62.40	62.50
	Total	69	24.48	12.58	6.10	62.50
K	Acacia	23	307	68	205	476
	Multifloral	44	731	397	333	2178
	Honeydew	2	2569	100	2498	2639
	Total	69	643	503	205	2639
Ca	Acacia	23	32.71	13.97	9.10	66.50
	Multifloral	44	146.82	59.67	56.10	300.10
	Honeydew	2	397.90	6.65	393.20	402.60
	Total	69	116.06	87.26	9.10	402.60
Mg	Acacia	23	7.27	2.06	3.90	10.60
	Multifloral	44	26.58	7.97	13.20	46.60
	Honeydew	2	64.25	1.06	63.50	65.00
	Total	69	21.24	13.43	3.90	65.00
Fe	Acacia	23	4.51	4.15	2.00	16.30
	Multifloral	44	7.19	7.50	2.00	35.10
	Honeydew	2	8.65	1.34	7.70	9.60
	Total	69	6.34	6.55	2.00	35.10
Cu	Acacia	23	0.67	0.41	0.17	1.79
	Multifloral	44	0.84	0.63	0.14	3.06
	Honeydew	2	1.94	0.09	1.87	2.00
	Total	69	0.81	0.59	0.14	3.06
Mn	Acacia	23	0.33	0.23	0.08	1.12
	Multifloral	44	0.48	0.19	0.18	1.17
	Honeydew	2	0.98	0.02	0.97	1.00
	Total	69	0.45	0.23	0.08	1.17

The pH is indeed a useful index of possible microbial contamination [38] and has high relevance during the extraction and storage of honey because it is related to the stability and the shelf life of the product [39]. As previously reported [40], most bacteria and moulds grow in a neutral and mildly alkaline environment respectively, while yeasts require an acidic environment (pH = 4.0 – 4.5) and do not grow in alkaline media. The analyzed honeys show a mean pH value of 3.70 with a range of between 3.50 – 4.21. The mean pH value of Marche honeys was lower than that reported by Conti (2000) [38] for Lazio (central Italy) honeys, by Downey *et al*. (2005) [41] for floral honeys collected in Ireland and by Serrano *et al*. (2004) [20] for Andalusia (Spain) honeys. The pH values showed a very good correlation with K levels in honeys (r = 0.766; *p *= 0.05).

Water content is strictly related to climatic conditions and the degree of maturity; anomalous values may be an index of adulterations. The water content generally depends on the botanical origin of the sample, the processing techniques and the storage conditions [38]. Mean humidity was 17.4 % with a range of between 15.1 – 21.0 %. Only 3 samples out of 69 showed levels of humidity slightly higher than the limit permitted by the Council Directive of 20% [1]. This confirms that the fermentation rate is very low in the analyzed samples. Reported data for humidity were very similar for the three honey types analyzed, showing very low SD levels (see table 1). Moisture values observed for Marche honeys were higher than those obtained for Lazio [38], Andalusia [20] and Greece [12] honeys, but similar results were found by Downey *et al*. (2005) [41] for Ireland unifloral honeys.

The average sugar content was 81.16 % and the range was 77.60 – 83.80 %. Sugar content showed normal levels similar to those reported for Spanish thyme honeys [39]. Sugar and moisture content, as previously reported for Lazio honeys, are strictly correlated [38]. This study confirms the very good correlation value (r = 0.996; p = 0.01) between these quality parameters.

Mean mineral contents were (μg g^-1 ^dry wt.): Na, 24.5; K, 643; Ca 116; Mg, 21.2; Cu, 0.81; Fe, 6.34; Mn, 0.44. The mean fresh weight/wet weight ratio was 1.21 (n = 69). For data comparison, the reported results were appropriately transformed (i.e. wet or dry basis) when necessary.

Potassium, which accounts for 79% of the total mineral content, was quantitatively the most abundant of the elements present. This result is consistent with other reported data [42,43].

Our mean K levels were higher than for Lazio honeys [40,38] and mean levels in Morocco honeys [44], but smaller than those reported for Spanish honeys collected from different regions [45] and Slovenian honeys [46].

The mean sodium content (24.5 μg g^-1 ^) was significantly lower than in Lazio [38] and Spanish honeys [45], whose contents were 80.0 and 75.7 μg g^-1 ^d.w. respectively.

Magnesium levels (21.24 μg g^-1 ^) were lower than in Lazio [38], Morocco [44] and Spanish honeys [45], that were 30.85, 32.05 and 38.98 μg g^-1 ^respectively, but higher than those for Turkish honeys [47].

Calcium levels (116.1 μg g^-1 ^) were significantly higher than for Lazio honeys [38] and Slovenian honeys [46]. Moreover, the levels of Ca reported here are lower than for Spanish honeys [45] that was 168.8 μg g^-1 ^.

The mean iron level in Marche honeys (6.34 μg g^-1 ^) was significantly higher than for Lazio [38] and Turkish honeys [47] and lower than for Morocco honeys [44].

The mean cooper level for the Marche honeys (0.81 μg g^-1 ^) is very similar to that reported by Terrab *et al*. (2003) [44] and Fernàndez Torres *et al*. (2005) [45]. The mean manganese level (0.45 μg g^-1 ^) was lower with respect to those found to Lazio [38], Morocco [44], Spanish [45] and Slovenian honeys [46].

Potassium showed positive correlation with Ca (r = 0.645), Mn (r = 0.670) and Mg (r = 0.759). A very high positive correlation was also found between Ca and Mg, that is, r = 0.928. Moreover, Na correlated with Ca (r = 0.825) and Mg (r = 0.826).

From the results of the Kolmogorov test, the distributions within each honey type can be considered normal (*p-value *< 0.05), but the Levene test of the homogeneity of variances shows that there are differences among the factor levels (honey types) for some variables. For this reason, Welch's robust test for the equality of means was conducted.

The one-way ANOVA (table 2), which considered floral origin as main factor, shows that statistically significant differences were found for all studied parameters with the exception of sugar, Fe, and moisture. Thus, these variables were not considered in the application of LDA. These results showed that moisture is not associated with the botanical origin of honeys, as also reported by other authors [20,22,48]. Contrarily, some studies have reported that moisture is related with botanical origin [35,37].

**Table 2:** Equality of means tests.

	Anova test		Welch test	
	F	Sig.	Statistic	Sig.

Sugar	1.738	0.184	2.011	0.281
pH	15.001	0.000	55.083	0.003
Na	57.273	0.000	1396.299	0.000
K	49.602	0.000	411.465	0.000
Ca	75.501	0.000	1907.323	0.000
Mg	110.314	0.000	1887.915	0.000
Fe	1.404	0.253	4.817	0.059
Cu	4.788	0.011	81.891	0.000
Mn	10.721	0.000	179.333	0.000
Moisture	1.656	0.199	2.018	0.259

Table 3 shows the factor loading obtained for the first two factors and the variance explained by each of them. The first two principal components accounted for more than 75% of the variation in the honey samples analysed. The first principal component (PC1) explains 59.8% of the variance, and the second (PC2) explains 17.3% of the variance. According to the loading matrix (table 3), it can be observed that Mg and K are the dominant parameters in the first factor, while Ca, Na and Mn showed slightly lower values. Similar results, for K and Mg, were reported by other authors [44,45]. The factor loading in PC2 showed that pH resulted the most dominant variable in this PC.

**Table 3:** Principal component analysis (PCA). Loadings of the variables, eigenvalues, explained and cumulative variance for the first two first PCs.

	Variance Explained			Factor loading						
	
PC	Eigenvalues	% of Variance	Cumulative %	pH	Na	K	Ca	Mg	Cu	Mn
1	4.189	59.841	59.841	0.471	0.845	0.906	0.888	0.948	0.435	0.746
2	1.214	17.337	77.178	0.719	-0.369	0.216	-0.363	-0.215	0.575	0.073

**Figure 1:**
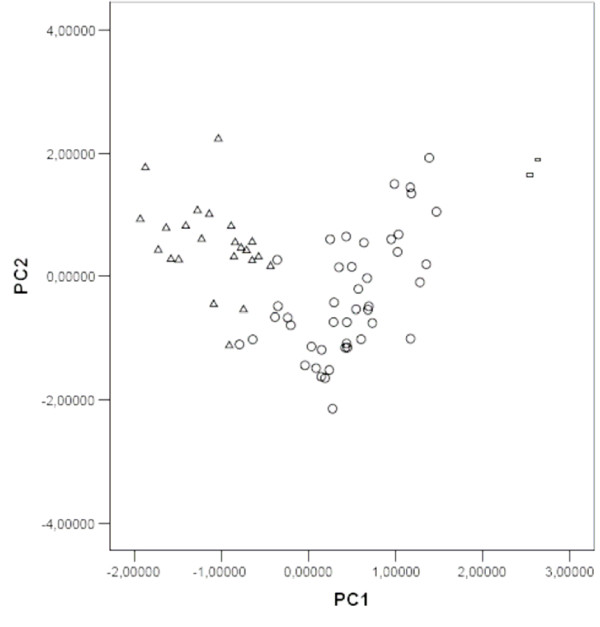
Principal component score plot. (Botanical origins: △ Acacia, ◯ Multifloral, ▱ Honeydew).

Examining the graphical distribution of the honey samples on the reported plot (figure 1) using the PC1 and PC2 principal components as coordinate axes, a natural separation of the three honey groups of different botanical origin was found. However, some multifloral and acacia honey samples did overlap. PCA results suggested that physicochemical parameters and mineral component data could provide useful information to achieve a botanical classification for the investigated honey.

Wilks's lambda test (table 4) shows that each discriminant function is significant (p-value < 0.05) thus allowing each to be used for model interpretation. Table 4 also shows the eigenvalues, the percentage variance explained by each function, the cumulative percentage variance explained and canonical correlation (R). These results shows that the first discriminant function is the most important in honey sample classification.

**Table 4:** Tests of significance, eigenvalues and canonical correlation for the discriminant functions.

	Test of Wilks' Lambda				Eigenvalues			
	
Function	Wilks' Lambda	Chi-square	df	p-value	Eigenvalue	% of Variance	Cumulative %	Canonical Correlation
1	0.085	155.110	14	0.000	6.219	90.9	90.9	0.928
2	0.616	30.573	6	0.000	0.625	9.1	100.0	0.620

The standardized discriminant coefficients (table 5) are used to compare the relative importance of the independent variables, for instance, beta weights are used in regression [49,50]. The higher the absolute value of a standardized coefficient, then the more significant is the related selected variable in the canonical variable. Mg resulted in being the parameter that contributes most to the first canonical variable (standardized coefficient = 0.893), accounting for most of the discrimination between honey classes (~91%) while K and pH show lower values.

**Table 5:** Standardized coefficients for canonical variables obtained by discriminant analysis.

	Standardized Discriminant Coefficients	
	
	Function	
	
	1	2
pH	-0.651	1.037
Na	0.158	0.492
K	0.631	0.172
Ca	-0.054	0.619
Mg	0.893	-1.001
Cu	0.003	0.209
Mn	-0.367	0.037

For DF1, Mg is the most important variable in explaining the separation in the honey samples according to botanical origin. The second canonical variable is related positively to pH and negatively to Mg, as deduced from the high absolute values of the standardized coefficients (1.04, and -1.0, respectively). This explains more than 9% of the variance. The loading in DF2 shows that pH is the most important variable in explaining the separation between honey samples. In fact, pH has been previously described as a possible indicator of the botanical origin for honeys [22,37,48].

**Figure 2:**
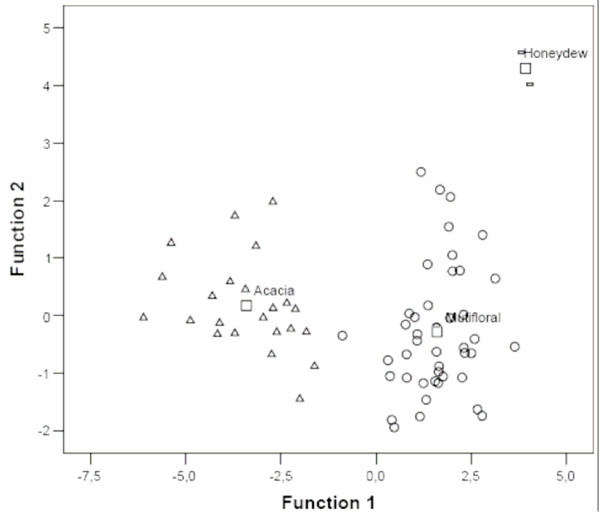
Canonical plots: honeys are located in the space formed by two discriminant functions (Botanical origins:  △ Acacia, ◯ Multifloral, ▱ Honeydew, □ Group centroid).

The scatter diagram of honey samples, the axes of which are the first two canonical variables (figure 2), shows that three types of honeys appear completely separated in the plot.

LDA can be also used to predict the group membership of honeys. The results of classification, when all the samples were in the training set, are shown in table 6. It reports the number (and percentages) of samples correctly classified into each honey type (on the diagonal of the matrix) and those that were misclassified. The LDA total error of classification was very low (0.8%).

**Table 6:** Classification results of LDA of seven variables (pH, Na, K, Ca, Mg, Cu, Mn) in multifloral and unifloral Marche honeys.

Original					
		Predicted Group Membership			

	Botanical Origins	Acacia	Mutifloral	Honeydew	Total
Count	Acacia	23	0	0	23
	Mutifloral	0	44	0	44
	Honeydew	0	0	2	2
%	Acacia	100.0	0	0	100.0
	Mutifloral	0	100.0	0	100.0
	Honeydew	0	0	100.0	100.0

Cross-validated method					

		Predicted Group Membership			

	Botanical Origins	Acacia	Mutifloral	Honeydew	Total
Count	Acacia	23	0	0	23
	Mutifloral	1	43	0	44
	Honeydew	0	0	2	2
%	Acacia	100.0	0	0	100.0
	Mutifloral	2.3	97.7	0	100.0
	Honeydew	0	0	100.0	100.0

All acacia, honeydew, and multifloral honey samples were correctly classified into their *a priori *established honey types. Generally, it is not difficult to obtain very good classification if the same cases are used for the model estimation. In order to have a more exact idea of the forecast LDA performance, it is more useful to classify cases that were not previously used for the estimation of the LDA model, such as cross-validation methods.

The "leave-one-out" method [51] was performed. This method classifies a particular sample by considering the whole set of samples but excluding the contribution of the sample being classified.

Table 6 shows the results of this study. Acacia and honeydew honey samples were correctly classified in their *a priori *established honey types (100%), while multifloral honeys show slightly lower agreement percentages (97.7%).

In conclusion, the analytical results obtained for the Marche honeys indicated a good level of quality of this product. The determination of physicochemical parameters and mineral content in combination with modern statistical techniques is a useful tool for honey classification. In this study PCA explained more than 75% of the variance with the first two PC variables. The variables with higher discrimination power, according to the multivariate statistical procedure, were Mg and pH. On the other hand, all samples of acacia and honeydew, and more than 90% of samples of multifloral type have been correctly classified by using the LDA.

## Conclusion

In general, the analytical results obtained for the Marche honeys indicate the products' high quality. The determination of physicochemical parameters and mineral content in combination with modern statistical techniques can be a useful tool for honey classification. However, more studies are needed in order to characterize unifloral and multifloral honeys by means of pattern recognition methods of zones of relevant honey production.

## Experimental

### Samples

The study was conducted on 69 samples of the typical honeys produced in the Marche Region in central Italy: 44 multifloral, 23 acacia, 2 honeydew. All collected samples were taken from the local beekeepers' association with a guarantee of genuineness. All samples were collected and stored in holders and immediately transferred to the laboratory where they were kept at 4–5°C until analysis.

### pH, sugar content and moisture

The pH was assessed by means of a potentiometer utilizing a pH meter Mettler Delta 345 (Mettler Toledo, Milano, Italy) [52]. Sugar and moisture values were determined utilizing a special refractometer Bertuzzi (Bertuzzi, Milano, Italy) owing two direct reading displays, for the measurement of sugar content and moisture percent respectively (Chatway method). Sugar content was expressed as brix degrees [52].

### Determination of mineral elements

About 0.6–0.7 g of fresh honey was treated with 8 ml of 70 % (w/w) Nitric Acid Suprapur (Merck, Suprapur, Darmstadt, Germany) and 2 ml of 30 % (w/w) Hydrogen Peroxide Suprapur (Merck, Darmstadt, Germany) in PTFE vessels. The microwave closed digestion system (MDS 2000, CEM Corporation, North Caroline, USA) was used for the mineralization process. The treatment procedure was programmed in five steps with a power of 600 W applied for 5 min at each; the pressure in the system was set as follows: 20, 40, 85, 140 and 200 psi. Subsequently, digestion vessels were cooled to room temperature. The final clear solution was made up to 50 mL with DWI water. Simultaneously, duplicate digestion blanks were prepared.

All mineral elements in digested solutions were determined using a Shimadzu 6800 Atomic Absorption Spectrometer (Kyoto, Japan) coupled to different atomic vapor generators depending of analytical concentration. A graphite furnace accessory GFA-6000 and autosampler ASC-6000 were employed for Cu and Mn measurements and a flame of air/acetylene was used for Fe, Ca, Mg, Na and K.

All chemicals used in sample treatments were ultra-pure grade (HNO_3_, H_2_O_2 _30%, Merck, Suprapur, Darmstadt, Germany). Ultra-pure water (Milli-Q system, Millipore Corporation, U.S.A.) was used for all solutions. All glassware was cleaned prior to use by soaking in 10 % v/v HNO_3 _for 24 hours before rinsing with Milli-Q water. The standard metal solutions were prepared from stock standard solutions of ultra-pure grade supplied by Merck (Darmstadt, Germany).

The traceability of results was obtained from the analysis of the standard reference material NIST-1515 (apple leaves – National Institute of Standards and Technology) and the certified reference material Antartic Krill MURST-ISS-A2 (Italian Research Programme in Antártica). Table 7 shows the results obtained for Na, K, Ca, Mg. Cu, Fe and Mn in both materials. A sample of reference material and blanks was included in each analytical batch. Results were in very good agreement with certified values for all the elements considered proving good repeatability of the method employed.

**Table 7:** Results of the analysis of standard reference materials (μg g^-1 ^for Na, Cu, Mn, Fe and wt. percent for K, Ca, Mg, dry wt material)

Element	Apple leaves (SRM 1515, NIST)		Antartic krill (MURST-ISS-A2)	
	Found^a ^	Certified	Found^a ^	Certified

Na	25.8 ± 2.1	24.4 ± 1.2	-	-
K	1.65 ± 0.04	1.61 ± 0.02	-	-
Ca	1.47 ± 0.07	1.526 ± 0.015	-	-
Mg	0.259 ± 0.009	0.271 ± 0.008	-	-
Cu	5.34 ± 0.60	5.64 ± 0.24	63.1 ± 4.9	64.1 ± 5.1
Fe	89.6 ± 5.4	83 ± 5	60.3 ± 3.2	56.6 ± 2.8
Mn	52.1 ± 1.4	54 ± 3	3.99 ± 0.21	4.12 ± 0.16

^a ^Mean and standard deviation of replicates (n = 5)

### Statistical methods

The mean values of water content, pH, sugar and mineral concentration of the studied honeys (Acacia, Multifloral and Honeydew) were statistically compared by one-way analysis of variance (ANOVA) and the robust Welch test. Normality and homogeneity of variances in the data were verified using Kolmogorov-Smirnov and Levene tests. Bivariate correlations (by means of Pearson's correlation coefficient) between all considered parameters were studied in order to define which were of significance. Multivariate statistical techniques such as principal component analysis (PCA) and linear discriminant analysis (LDA) were used to determine the variables that better discriminate between honey types. The SPSS software version 13.0 and R 2.2.0 were used for all the chemometric calculations.

PCA is a classic technique to reduce the dimension of the initial data representing the original data matrix X as a product of two matrices, the score matrix and the loading matrix, by projecting the raw data onto a few-dimensional space (the principal components). Principal components (PCs) are not correlated and those that are first explain the major data variability [50,53]. The traditional approach is to use the first few PCs in data analysis since they capture most of the variation in the original data set. In this work PCA was used in order to visualize the relative distribution of the honey samples according to their botanical origin.

LDA is a widely used tool in pattern recognition. Given a nominal group variable and several quantitative attributes, the LDA extracts a set of linear combinations of the quantitative variables (called discriminant functions or canonical variables) that best reveal the differences among the groups by maximising the ratio of the sum of squares between-classes and the sum of squares within-classes [49]. The first discriminant function (*DF1*) extracted is that which separates the groups to a maximum. The second *DF*, orthogonal to the first, separates the groups based on variance not yet explained by the first *DF*. In this way their contributions to the discrimination between groups do not overlap. If the number of groups considered is *p*, there are *p *- 1 canonical variables that are orthogonal [49,54,55].

## Authors' contributions

MEC conceived of the study and, together with MBT, participated in its design and drafted the manuscript. MEC, MBT and JS coordinated the sampling protocols and the whole analytical procedures. DC participated in the design and performed the statistical analysis. This project was based on the ideas and under the guidance and consultation of MEC, MBT and LC. All authors read and approved the final manuscript.
